# Activity of Zn and Mg phthalocyanines and porphyrazines in amyloid aggregation of insulin

**DOI:** 10.1002/jmr.2660

**Published:** 2017-08-30

**Authors:** V. Kovalska, S. Chernii, M. Losytskyy, J. Ostapko, I. Tretyakova, A. Gorski, V. Chernii, S. Yarmoluk

**Affiliations:** ^1^ Institute of Molecular Biology and Genetics NASU Kyiv Ukraine; ^2^ V.I. Vernadskii Institute of General and Inorganic Chemistry NASU Kyiv Ukraine; ^3^ Institute of Physical Chemistry PAS Warsaw Poland

**Keywords:** amyloid fibrils, dynamic light scattering, fluorescence, phthalocyanines, porphyrazines, scanning electron microscopy

## Abstract

Formation of the deposits of protein aggregates—amyloid fibrils in an intracellular and intercellular space—is common to a large group of amyloid‐associated disorders. Among the approaches to develop of therapy of such disorders is the use of agents preventing protein fibrillization. Polyaromatic complexes—porphyrins and phthalocyanines—are known as compounds possessing anti‐fibrillogenic activity.

Here, we explore the impact of related macrocyclic complexes—phthalocyanines (Pc) and octaphenyl porphyrazines (Pz) of Mg and Zn—on aggregation of amyloidogenic protein insulin. Pz complexes are firstly reported as compounds able to affect protein fibrillization.

The effect of Pc and Pz complexes on the kinetics and intensity of insulin aggregation was studied by the fluorescent assay using amyloid sensitive cyanine dye. This has shown the impact of metal ion on the anti‐fibrillogenic properties of macrocyclic complexes—the effect on the fibrillization kinetics of Mg‐containing compounds is much more pronounced comparing to that of Zn analogues.

Scanning electron microscopy experiments have demonstrated that filamentous fibrils are the main product of aggregation both for free insulin and in the presence of macrocyclic complexes. However, those fibrils are distinct by their length and proneness to lateral aggregation. The Pc complexes cause the increase in variation of fibrils length 0.9 to 2.7 nm in opposite to 1.4 to 2.0 nm for free insulin, whereas Pz complexes cause certain shortening of the fibrils to 0.8 to 1.6 nm.

The averaged size of the fibrils population was estimated by dynamic light scattering; it correlates with the size of single fibrils detected by scanning electron microscopy.

## 1 INTRODUCTION

The formation of insoluble linear protein aggregates also known as amyloid fibrils is common to a large group of human diseases, including amyloidosis and neurodegenerative disorders. The list of these diseases includes more than 20 disorders such as neurodegenerative (Alzheimer's, Parkinson's diseases), hereditary amyloidosis, prion diseases, and type II diabetes mellitus.^1^, ^2^, ^3^ One of the important approaches to affect an amyloid formation process is the discovery of molecules with different structures potentially able to target this process.

Polyaromatic scaffolds belonging to chemical classes of porphyrins and phthalocyanines are widely studied as high potential anti‐amyloidogenic agents.^4^, ^5^, ^6^ Among such compounds, metal‐containing tetrasulfonated phthalocyanines (Figure S1 in Supporting Information) have been described as efficient inhibitors of α‐synuclein fibrillization.^5^ According to this study, the inhibitory activity noticeably depends on the central metal atom, the most pronounced effect was observed for Ni‐containing phthalocyanine with formation of small, amorphous aggregates and flat fibrils (in comparison with Al‐containing and Zn‐containing phthalocyanines as well as metal‐free ones (Figure S2 in Supporting Information).

Previously, we have discovered the anti‐fibrillogenic activity of axially coordinated phthalocyanines (Figure S3 in Supporting Information) that are the complexes with “spatial geometry” of the molecule.^7^, ^8^, ^9^ It has been shown that these phthalocyanine complexes are able to affect amyloid aggregation of proteins redirecting their fibrillization toward the formation of oligomeric aggregates or substantially inhibiting the formation of the fibrils.^7^, ^8^ The impact of such phthalocyanine on morphology of the formed amyloid aggregates largely depends on the nature of the out‐of‐plane ligand.

**Figure 1:**
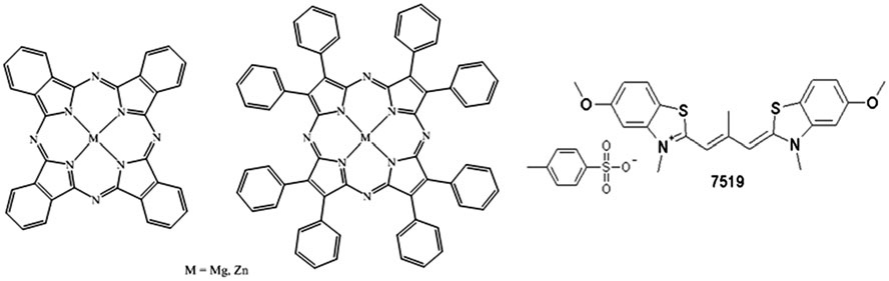
Structures of Mg and Zn phthalocyanines (PcMg, PcZn), octaphenylporphyrazines (PzMg, PzZn), and amyloid‐sensitive dye 7519 (from left to right)

In the present work, we examine the anti‐fibrillogenic activity of the structure‐related macrocyclic complexes Mg and Zn phthalocyanines and octaphenyl porphyrazines (Figure 1) using amyloidogenic protein insulin. The anti‐fibrillogenic properties of the compounds belonging to porphyrazines have been studied for the first time. The amyloid‐sensitive dye‐based assay is used to monitor the insulin fibrillization in real time for free protein and in the presence of the mentioned macrocyclic compounds that allows to estimate their effect on the efficiency and kinetics of the aggregation reaction. Scanning electron microscopy (SEM) and dynamic light scattering (DLS) are complementary methods used to study the morphology of the insulin fibrils: structures of the sole aggregates (SEM) and average size (dimensions) of the particles in population (DLS). Because those complexes contain the coordinatively unsaturated central metal ion (ie, the one able to easily form additional coordination bonds), its impact on interaction of macrocyclic complexes with protein is discussed.

## 2 MATERIALS AND METHODS

### 2.1 Materials

Mg and Zn phthalocyanines were prepared by metal template synthesis based on the corresponding metal alkanoates by methods described in Chernii et al^10^; octaphenylporphyrazines were synthesized using general methods described in Cook and Linstead.^11^

### 2.2 Insulin fibril formation

Human insulin was dissolved at 340 μM (2 mg/mL) concentration in 0.1 M water solution of HCl (pH 2) either in the absence (control solution) or in the presence of one of the inhibiting compounds under study (concentration of compounds was 100 μМ). Fibrils were formed by incubating the mentioned protein solutions in a thermo mixer incubator at 65°C for 5 hours. The fluorescence assay based on the cyanine dye 7519^12^ was used to monitor the amyloid aggregation degree. The presence and form of fibrillar aggregates were confirmed by SEM measurements.

### 2.3 7519 fluorescence assay

To investigate the kinetics of insulin fibril formation in the presence and in the absence of the studied compounds, 10‐μL aliquots were removed from the incubated mixture at various time points during the reaction and added to 2‐μM solution of the dye 7519 in 50 mM TRIS‐HCl buffer, pH 7.9. The fluorescence of 7519 was measured at λ_ex_ = 578 nm and λ_em_ = 588 nm. The percent of the fibrillogenesis suppression was calculated as [(1 − I / I_0_) × 100%], where I_0_ and I are the fluorescence intensity values of 7519 in the presence of insulin incubated in the absence and in the presence of the macrocyclic complex, respectively.

### 2.4 Scanning electron microscopy studies

SEM studies of the products of fibrillization reaction of insulin in the absence and in the presence of the studied compounds were carried out using FEI NovaNano 450 scanning electron microscope. For layer deposition, the samples of insulin amyloid aggregates at the concentration of 340 μM were diluted 15 times with distilled water. Then, a drop of the solution was applied to the special surface (ITO—tin‐doped indium oxide coated glass); the sample was studied after water evaporation. We use such parameters as 5 kV (useful for biological objects) and 5‐mm distance. The length of the fibrils was determined using Gwyddion program.

### 2.5 Dynamic light scattering

Buffers were filtered through a 0.2‐μM filter prior to use. Following cessation of fibrils, the intensity of light (λ = 632.8 nm) scattered from samples at the concentration of 27.2 μM of insulin fibrils was measured at 6 angles between 40° and 160° (40°, 60°, 90°, 120°, 140° for insulin amyloid fibrils in the presence of PcZn, PcMg, and PzMg; 40°, 60°, 90°, 120°, and 160° for free insulin amyloid fibrils and those obtained in the presence of PzZn). The sample was maintained at 25 ± 0.1°C using a circulating water bath to remove air bubbles. Experiments were performed using the Brookhaven Instruments analyzer. In DLS experiments, the normalized intensity time correlation function G^2^(q, τ) was obtained, q defined as q = (4πn_0_/λ) × sin(θ/2) being thus a function of the angle where n_0_ is a refraction index, and τ is a correlation time. For the obtained G^2^(q, τ) function, monoexponential fitting was performed by the dependence G^2^(q, τ) = A + B × e^−2Γτ^, Γ = Dq^2^ is the decay rate with D being the apparent translational diffusion coefficient. Results are presented as the dependence Γ/q^2^ on q^2^.^13^ Basing on D values, apparent hydrodynamic diameter of the fibrils can be estimated as d = kT/3πηD.

## 3 RESULTS AND DISCUSSION

### 3.1 Kinetics of insulin fibril formation monitored by amyloid sensitive dye

**Figure 2:**
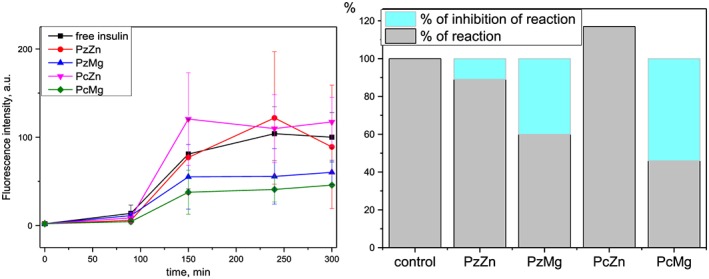
The effect of macrocyclic complexes on the kinetics and intensity of the insulin fibril formation monitored by fluorescent dye 7519. Left: The variation of fluorescence intensity of the dye with the path of the reaction. Right: Percentage of the insulin fibrils formed (gray) and changes in intensity of the fibril formation (cyan) at the end of the reaction. Experiments are performed 3 times. Standard deviation of the fluorescence measurement is represented as error bars

The influence of macrocyclic compounds on the kinetics of insulin fibrillization and degree of their inhibitory activity have been studied using fluorescent assay based on the amyloid‐sensitive dye 7519 (Figure 2). The dye specifically binds to the grooves of amyloid fibrils formed by the beta‐pleats and this way increases its emission intensity

The aggregation of amyloidogenic protein typically begins with a lag phase, when the formation and accumulation of the fibrillar seeds mainly occur and no significant increase in the fluorescence signal intensity is observed.^14^ Studied complexes do not change the duration of the lag‐phase of insulin fibrillization.

Mg‐containing compounds PcMg and PzMg essentially suppress the intensity of insulin fibrillization (up to 54% and 40% correspondingly at the end of the reaction).

Zn complexes have weaker effect on the insulin fibrillization as compared with Mg ones. PzZn suppresses insulin fibril formation by approximately 11% and slightly (by 17%) increases the reaction intensity. It is observed that the nature of metal ion affects the insulin fibril formation intensity stronger than the periphery arrangement of the macrocycle does.

We have examined whether the interaction of the reference dye 7519 with metal complexes could be responsible for the changes in the dye fluorescence intensity. It is shown that the addition of macrocyclic complexes slightly affects the fluorescence characteristics of the free dye 7519 or its complexes with insulin fibrils. We thus consider that the changes of the dye fluorescent response along the fibrillization reaction pathway are not determined by probable phthalocyanine‐dye interactions. Hence, the difference between the activities of Zn and Mg complexes shown by fluorescent assay is associated with their different behavior (supramolecular binding) in insulin aggregation reaction caused by the central metal atom effect.

### 3.2 Scanning electron microscopy study

**Figure 3:**
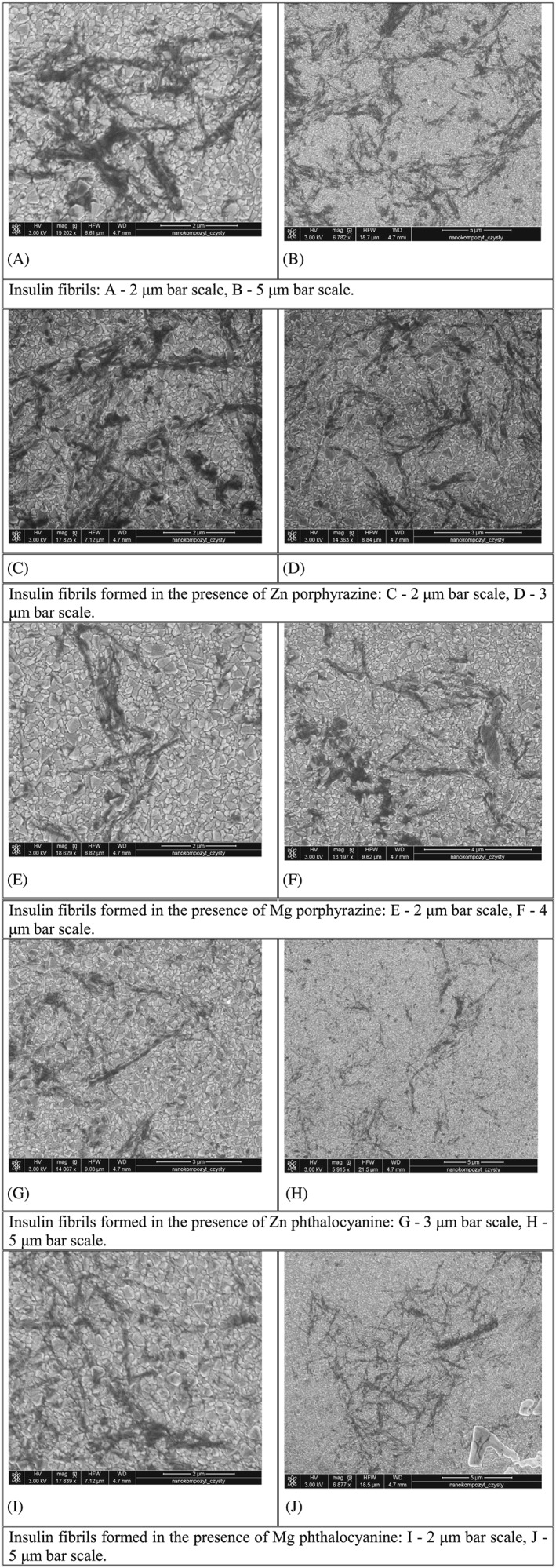
Scanning electron microscopy images of insulin fibrils formed in the absence and in the presence of investigated compounds

We study the impact of Mg and Zn phthalocyanines and porphyrazines on the morphology of amyloid aggregates of insulin by SEM (Figure 3).

Free insulin forms elongated filamentous species of the length 1.4 to 2 μm (Table 1) often with the branched structure. In addition, insulin fibrils have a proneness to lateral aggregation and thus form the so‐called bundles^15^ (Figure 3A,B). The high density of the fibrils and their bundles on the image may point on high intensity of the fibrillization reaction.

**Table 1:** Length (L) of fibrillar insulin (fINS) obtained by SEM and apparent hydrodynamic diameter (d) of fINS calculated by DLS in the absence and in the presence of porphyrazines and phthalocyanines

Sample	Free fINS	In the presence of PzZn	In the presence of PzMg	In the presence of PcZn	In the presence of PcMg
L, μm	1.4–2.0	0.8–1.6	0.6–1.3	0.9–2.7	1.2–2.3
d, μm	1.0 ± 0.3	1.2 ± 0.3	1.7 ± 0.3	2.9 ± 0.7	1.7 ± 0.4

L, fibril length, determined basing on SEM images (presented in Figure 3 and not presented ones); d, apparent hydrodynamic diameter.

Previously, we have reported the ability of phthalocyanines with out‐of‐plane ligands to redirect the insulin fibrillization toward the formation of different kinds of aggregates (oligomeric or amorphous species) or cause changes of morphology of fibrils.^7^, ^8^ In the presence of earlier studied Hf phthalocyanine bearing quinolinium styryl ligand, the thinning and elongation of the fibrils are observed.^16^ Influence of the studied there macrocyclic compounds on the morphology of the formed insulin aggregates is less significant.

It is seen from the Figure 3 that in the presence of macrocyclic compounds the filamentous aggregates remain the main product of the fibrillization reaction.

The insulin aggregates formed in PzZn presence are elongated filaments (Figure 3C,D) with the length in the range 0.8 to 1.6 μm (Table 1) of the strongly branched structure and proneness to lateral aggregation. In the case of PzMg presence, insulin forms fibrils with the length of approximately 0.6 to 1.3 μm (Figure 3E,F). For fibrils induced by the presence of PzMg, lateral aggregation is less pronounced comparing with free insulin or PzZn. Generally, the insulin fibrils obtained in the presence of porphyrazines are of the smaller length than those of free insulin.

The effect of PcZn results in the increase of the dispersion of the fibrils length 0.9 to 2.7 μm (Figure 3G,H) comparing with free insulin (Figure 3A,B). The essential dispersion of the fibrils length is observed in the presence of PcMg—fibril length is approximately 1.2 to 2.3 μm (Table 1); moreover, a huge rod‐like aggregate with the size of 4.3 μm is formed (Figure 3J).

Generally, in the presence of phthalocyanines comparing with free protein, the larger quantity of single long fibrils is formed (less tendency to lateral aggregation).

Pc and Pz complexes differently affect the morphology of insulin aggregates, ie, they induce the formation of fibrils with different average length, “thickness,” and tendency to sticking together. This could be explained by different “geometry” of the complexes. Phthalocyanines are planar molecules, while porphyrazines have planar macrocyclic core with non‐planar arrangement on periphery provided by phenyl moieties (that are able to twist relatively to the core plane). Thus, steric hindrances are caused by phthalocyanines and porphyrazines upon their supramolecular binding to polypeptide chains, and they change the growth of the fibrils and affect their morphology in different ways.

### 3.3 Dynamic light scattering study

**Figure 4:**
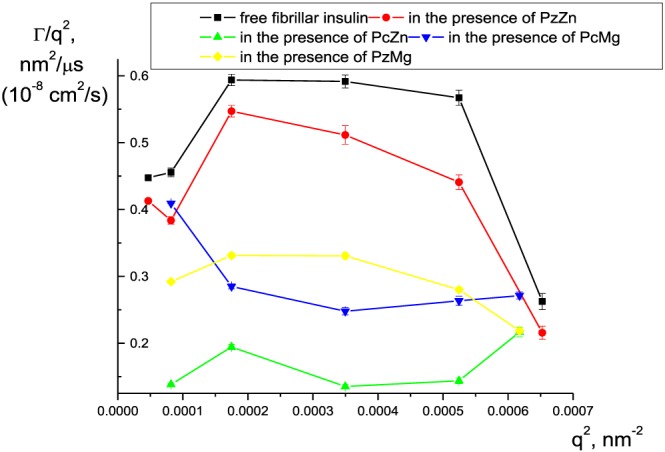
Dynamic light scattering measurements of insulin amyloid fibrils size. Dependence of (Γ)/q^2^ on q^2^ is presented for 27.2 μM of fibrils of free insulin (black line) and those formed in the presence of PzZn (red line), PcZn (green line), PcMg (blue line), and PzMg (yellow line)

Dynamic light scattering measurements point on distribution of hydrodynamic parameters of aggregates formed in the presence of the studied compounds. The data obtained at different values of the scattering angle are presented as plots of Γ/q^2^ on q^2^ (see subsection 2.5) in Figure 4. Because of intrinsic heterogeneity of fibrils in their suspension, there is typically a distribution of particle shapes, sizes, and thus hydrodynamic radius values.^17^ It should be noted that fibrillar structures are not trivial to study by light scattering even in stationary solutions.^18^ In our case, the solutions of fibrils with different shapes (separate fibrils, branched fibrils, or mature fibrils clusters) and sizes are characterized by estimated apparent hydrodynamic radius based on the sphere approximation. The values of apparent hydrodynamic radius for the fibrils can reach up to several micrometers due to a rod‐like fibrils morphology.^19^

Because we have shown by SEM the formation of fibrils of similar morphology in the presence of all complexes, we also compare the apparent hydrodynamic diameter of these fibrils obtained by DLS. For this purpose, the apparent diffusion coefficients are estimated from the plots of Γ/q^2^ on q^2^ and are then converted to an apparent hydrodynamic diameter (Table 1). Hydrodynamic diameters estimated by DLS generally correlate with fibril size obtained by SEM. This calculation gives the smallest *d* values for free fibrillar insulin (approximately 1.0 ± 0.3 μm) and the fibrils formed in the presence of PzZn (approximately 1.2 ± 0.3 μm). The highest apparent hydrodynamic diameter value is obtained in the case of PcZn (the average value is equal to 2.9 ± 0.7 μm). This result correlates with the SEM data: because larger particles are known to make non‐proportionally higher contribution into the DLS intensity, fibrils with the highest upper limit of SEM size (Table 1) have also the highest apparent hydrodynamic diameter. As for fibrils induced by the presence of Mg phthalocyanine and Zn porphyrazine, they have shown similar values of hydrodynamic diameter (1.7 ± 0.4 and 1.7 ± 0.3 μm).

### 3.4 Discussion of the impact of central metal atom on anti‐fibrillogenic activity of macrocycles

The fluorescent dye‐based assay has shown the noticeable difference between the effect of the Zn and Mg complexes (in pair PzMg, PzZn and in pair PcMg, PcZn) on the intensity of insulin aggregation. However, the SEM reveals that morphology of the formed fibrils does not depend from the nature of the central metal atom. That is, it is the same for both Pc‐induced fibrils, as well as it is the same for both Pz‐induced ones.

Studied macrocyclic complexes (in corresponding pairs) have close physico‐chemical properties, ie, solubility, self‐association proneness, and coordination ability of central metal ions. Thus, we explain distinctions in the effect of Zn and Mg complexes on fibrillization intensity by the ability of Zn ion to coordinate histidine and cysteine aminoacid residues in proteins.^20^

**Figure 5:**
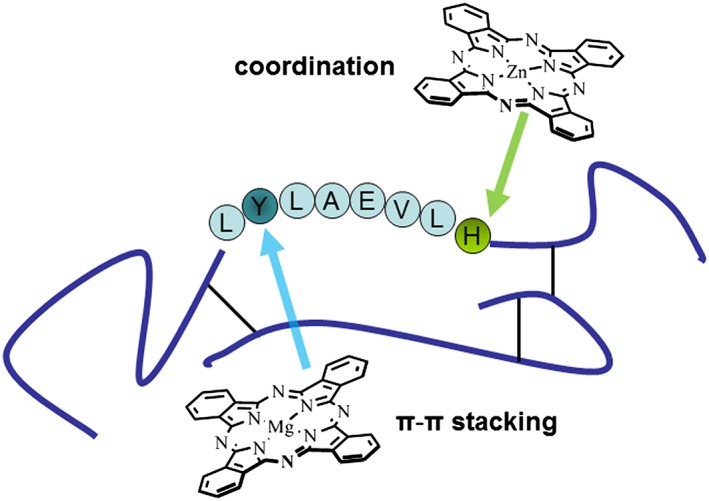
The scheme of human insulin, which represents an amyloidogenic sequence in B‐chain, a location of tyrosine residue Y16 and histidine residue H10 that are suggested as possible binding place for Mg and Zn complexes, respectively

In insulin, the histidine residue H10 (Figure 5) is involved in hexamer formation by Zn^+2^ coordination.^21^ This H10 is located near the amyloidogenic sequence (11)LVEALYL(17) in insulin B‐chain, which is responsible for the formation of fibril core.^22^

Generally, the structural basis for the effect of macrocyclic compound on amyloid fibril formation relies on specific π‐π interactions between the aromatic ring system of these molecules and aromatic residues of protein.^4^ However, we suppose that due to the mentioned “affinity” of Zn to histidine residue, Zn and Mg complexes could prefer different sites into the insulin amyloidogenic region for their binding. In the case of Mg‐containing complex, it preferably stacks to tyrosine residue inside the amyloidogenic region of insulin that forms the core of the fibril. Zn‐containing complexes predominantly bind on the periphery of this core due to their coordination to histidine residue. In this case, Mg complexes could suppress the formation of insulin fibrils with higher efficiency as compared with Zn complexes that results in lower intensity of the fluorescent response of amyloid sensitive dye.

Also, we could suggest that due to its location in fibrillar core, the molecule of Mg complex hinders the binding of the dye more strongly than the molecule of Zn complex placed out of the core. That would provide additional decrease of the dye fluorescent response in the case of Mg complexes.

## 4 SUMMARY

The effect of macrocyclic complexes Mg, Zn porphyrazines, and phthalocyanines on the insulin fibrillization has been studied using fluorescent spectroscopy, SEM, and DLS techniques. Anti‐fibrillogenic properties of porphyrazines are explored for the first time.

According to the fluorescent assay, Mg‐containing compounds reduce the intensity of fibrillization reaction (54% for Mg phthalocyanine and 40% for Mg porphyrazine) more strongly than corresponding Zn‐containing analogues (up to 17%). Hence, the nature of central metal ion could noticeably impact the anti‐fibrillogenic activity of macrocyclic complexes. Here, it is explained by the strong tendency of Zn ions to coordination with histidine residues.

SEM images demonstrate that insulin forms predominantly filamentous aggregates when free and in the presence of studied complexes. Fibrils of free insulin are 1.4 to 2 μm long with intense tendency to lateral aggregation. In the presence of porphyrazines, the shorter fibrils with the length of 0.6 to 1.6 μm grew. In the case of phthalocyanines, the high dispersion of the length of the fibrils (0.9–2.7 μm) is observed.

Average dimensions in fibril populations estimated by DLS correlate with the size of the corresponding single fibrillar species determined by SEM.

## Supporting information

Figure S1 Metal‐containing tetrasulfonated phthalocyanines
Figure S2 Metal‐free tetrasulfonated phthalocyanines
Figure S3 Axially coordinated phthalocyanines
