# Supplementary material for: Activity of Zn and Mg phthalocyanines and porphyrazines in amyloid aggregation of insulin
Source: J Mol Recognit. 2017 Aug 30;31(1):e2660. doi: 10.1002/jmr.2660 (PMC6175167; doi:10.1002/jmr.2660)
Supplement: Supplementary file 1 — Figure S1 Metal‐containing tetrasulfonated phthalocyanines Figure S2 Metal‐free tetrasulfonated phthalocyanines Figure S3 Axially coordinated phthalocyanines [file JMR-31-na-s001.doc]

Fig. 1 Metal-containing tetrasulfonated phthalocyanines

**
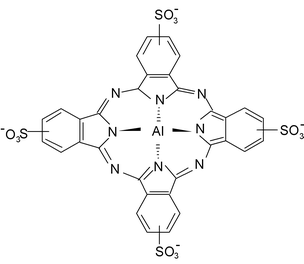
**

Fig. 2 Metal-free tetrasulfonated phthalocyanines

**
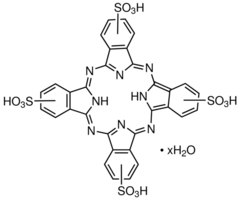
**

Fig. 3 Axially coordinated phthalocyanines
